# Endoscopic resection of acetabular screw tip to decompress sciatic nerve following total hip arthroplasty

**DOI:** 10.1186/s12891-018-2091-x

**Published:** 2018-06-04

**Authors:** Sun-jung Yoon, Myung-sik Park, Dean K. Matsuda, Yun Ho Choi

**Affiliations:** 1 Department of Orthopedic Surgery, Research Institute of clinical medicine of Chonbuk National University, Biomedical Research Institute of Chonbuk National University Hospital, 54907 Gunji-Ro 20, Dukjin-Gu, Chonbuk, Jeonju, South Korea; 2 DISC Sports and Spine Center, Marina del Rey, CA USA; 3 Department of Anatomy, Medical School, Institute for Medical Sciences, Chonbuk National University, Jeonju, 561-180 South Korea

**Keywords:** Endoscopic sciatic nerve decompression, Sciatic nerve neuropathy, Acetabular dome screw, Total hip arthroplasty

## Abstract

**Background:**

Sciatic nerve injuries following total hip arthroplasty are disabling complications. Although degrees of injury are variable from neuropraxia to neurotmesis, mechanical irritation of sciatic nerve might be occurred by protruding hardware. This case shows endoscopic decompression for protruded acetabular screw irritating sciatic nerve, the techniques described herein may permit broader arthroscopic/endoscopic applications for management of complications after reconstructive hip surgery.

**Case presentation:**

An 80-year-old man complained of severe pain and paresthesias following acetabular component revision surgery. Physical findings included right buttock pain with radiating pain to lower extremity. Radiographs and computed tomography imaging showed that the sharp end of protruded screw invaded greater sciatic foramen anterior to posterior and distal to proximal direction at sciatic notch level. A protruding tip of the acetabular screw at the sciatic notch was decompressed by use of techniques gained from experience performing endoscopic sciatic nerve decompression. The pre-operative pain and paresthesias resolved post-operatively after recovering from anesthesia.

**Conclusions:**

This case report describes the first documented endoscopic resection of the tip of the acetabular screw irritating sciatic nerve after total hip arthroplasty. If endoscopic resection of an offending acetabular screw can be performed in a safe and minimally invasive manner, one can envision a future expansion of the role of hip arthroscopic surgery in several complications management after total hip arthroplasty.

**Electronic supplementary material:**

The online version of this article (10.1186/s12891-018-2091-x) contains supplementary material, which is available to authorized users.

## Background

Sciatic nerve irritation due to an acetabular screw following total hip arthroplasty (THA) is rare; the few reported were treated with revision procedure to remove an acetabular screw [1]. Recent reports of the orthopaedic literature document the use of endoscopic decompression of sciatic nerve entrapment syndrome/deep gluteal syndrome treatment [2]. Endoscopy allows for complete extrapelvic sciatic nerve visualization and safe nerve decompression in the deep gluteal space [3, 4]. This is the first documented case of completely endoscopic treatment of sciatic nerve irritation by a protruded acetabular dome screw following THA.

## Case presentation

**Fig. 1:**
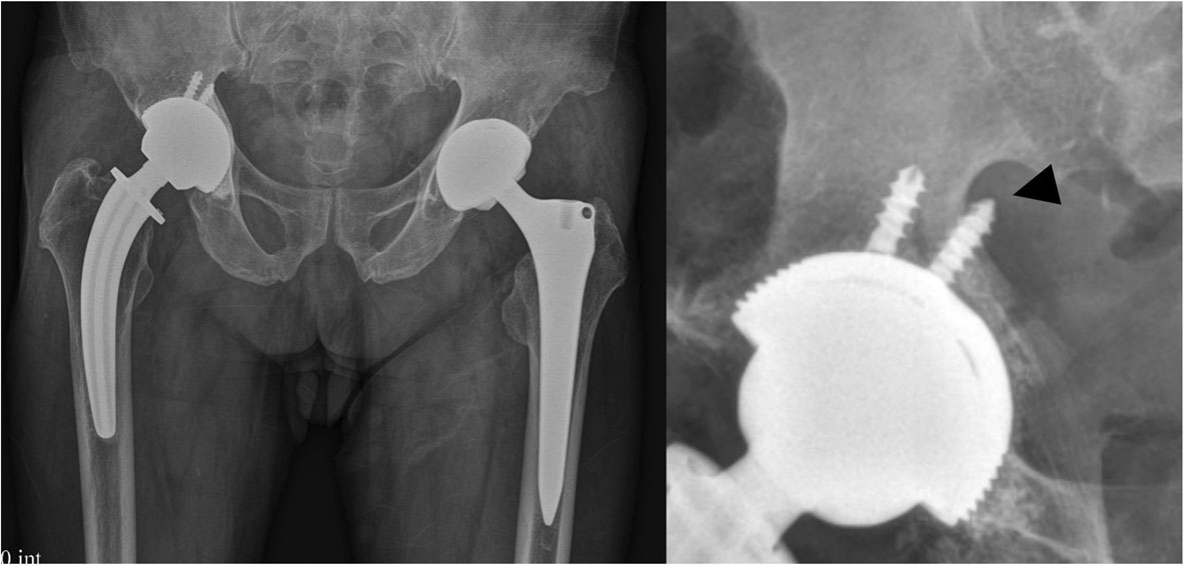
Preoperative hip radiographs (anteroposterior view; Left, iliac oblique view; Right) with protruded dome screw (arrowhead) into sciatic notch

**Fig. 2:**
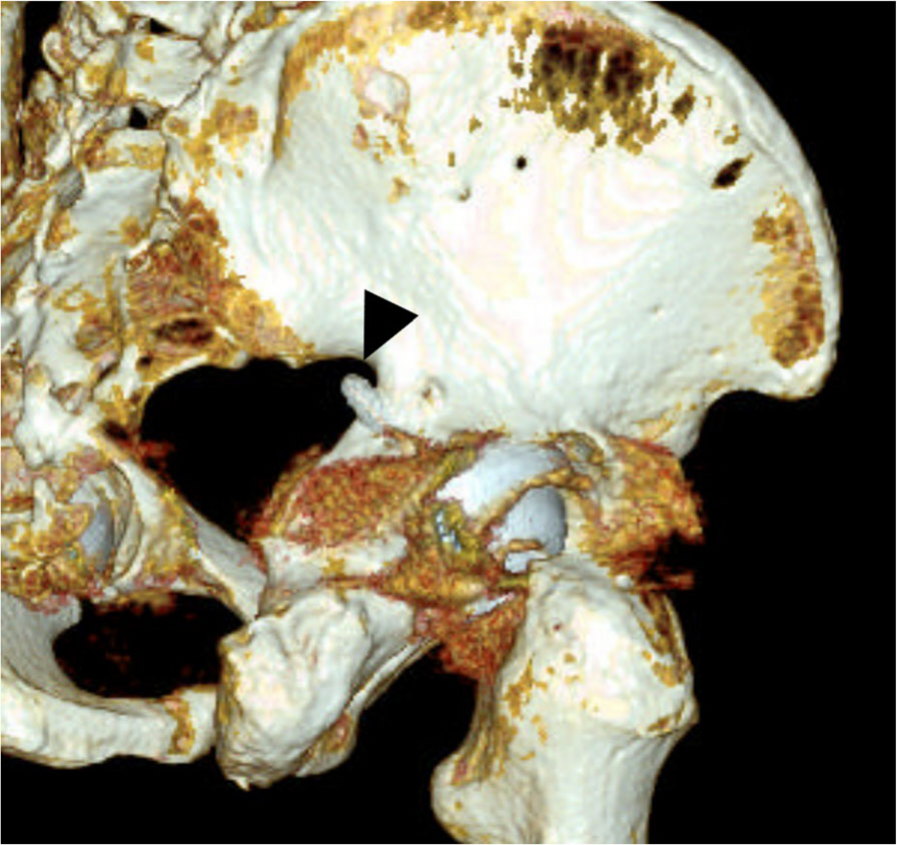
Oblique 3-dimensional computed tomography reconstruction showing an acetabular screw protruded into sciatic notch (arrowhead)

**Fig. 3:**
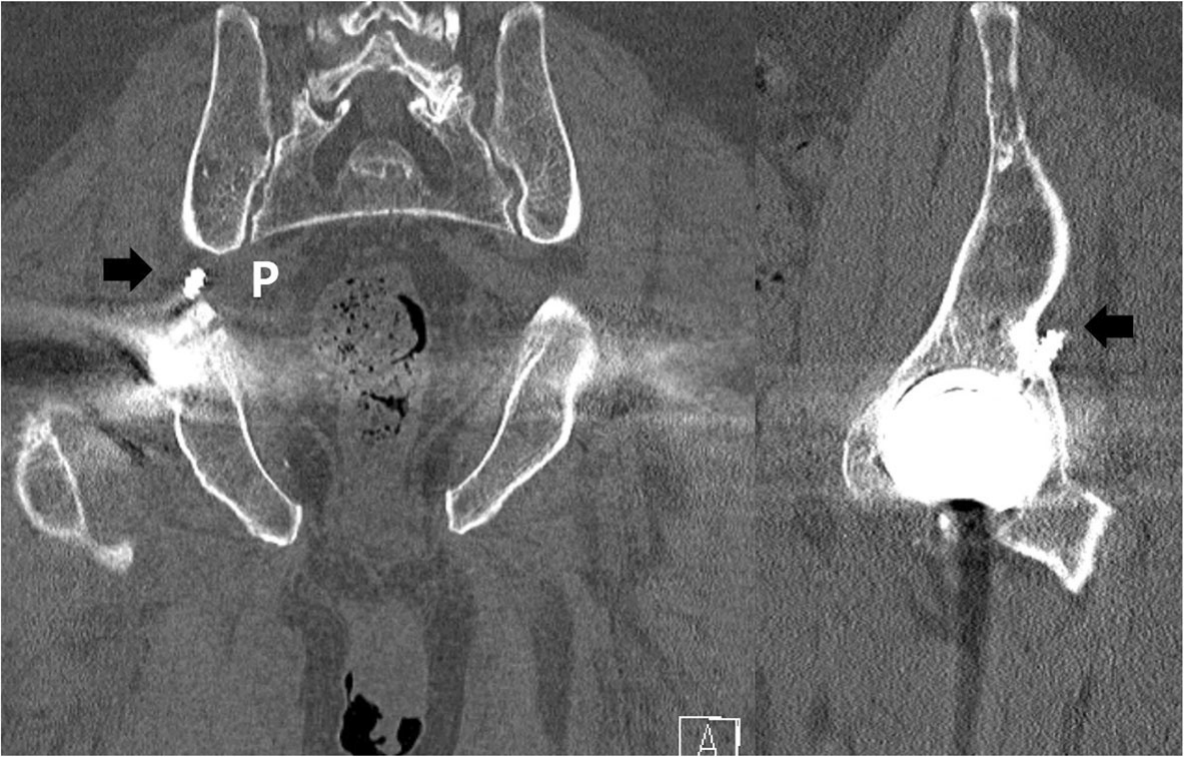
Coronal and computed tomography views of right hip showing sciatic notch area occupied by an acetabular screw (arrow). The protruded screw was placed superiorly than piriformis muscle (P)

**Fig. 4:**
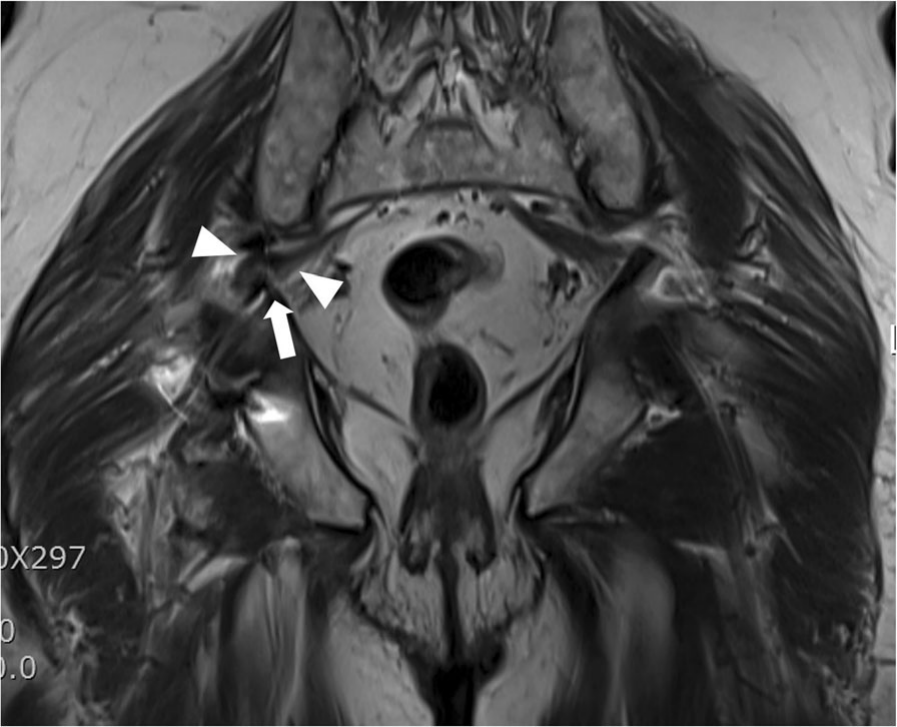
T2 turbo spin echo magnetic resonance image displaying a screw protrusion (arrowhead) and irritated piriformis muscle (arrow)

An 80-year-old man complained of severe pain and paresthesias following acetabular component revision surgery via the original posterolateral approach. The patient subsequently complained of right leg pain and paresthesias. The symptoms were exaggerated during initial hip flexion and internal rotation that limited ambulation. Physical findings included right buttock pain with radiating pain to the ipsilateral lower extremity. Radiographs and computed tomography imaging (Figs. 1, 2, 3, and 4) showed an acetabular screw tip protruding into the greater sciatic foramen in an anterior to posterior and distal to proximal direction at the level of the sciatic notch. The screw was 30 mm in length and positioned at posterior-inferior acetabular quadrant. Magnetic resonance imaging demonstrated tenting of the right piriformis muscle tented by the end of screw. Mechanical irritation of the sciatic nerve screw was suspected.

**Fig. 5:**
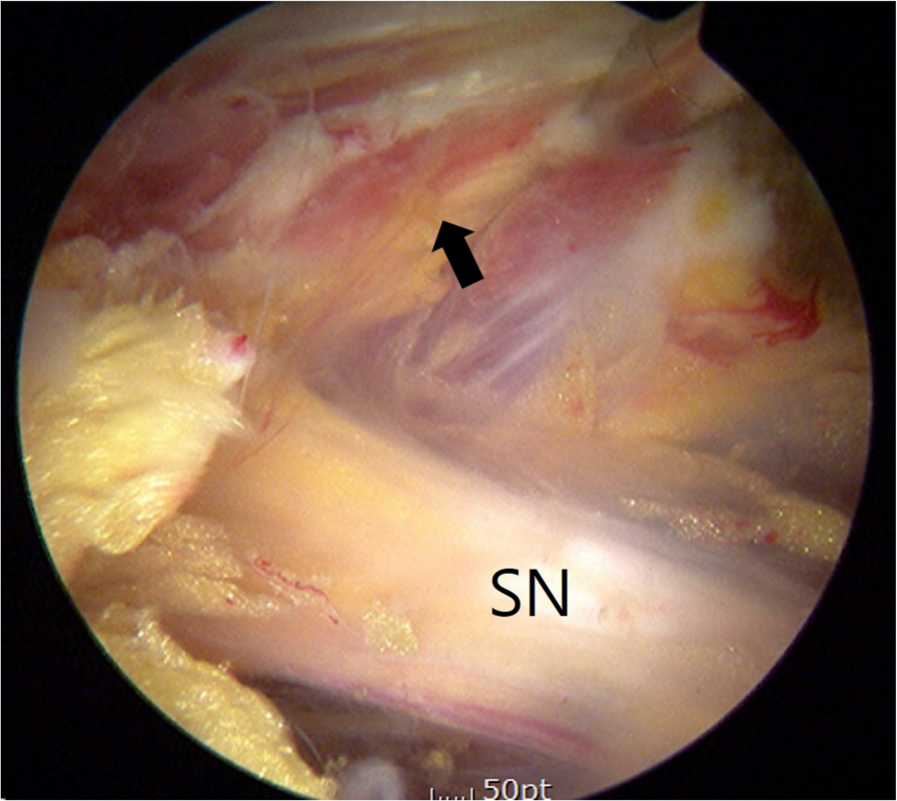
Endoscopic view from anterolateral portal of right hip (supine, upward tilt ipsilateral hip) showing repaired piriformis in previous surgery (arrow). Sciatic nerve (SN) is pale and loss of perineural fat. It is better visualized in the accompanying video

**Fig. 6:**
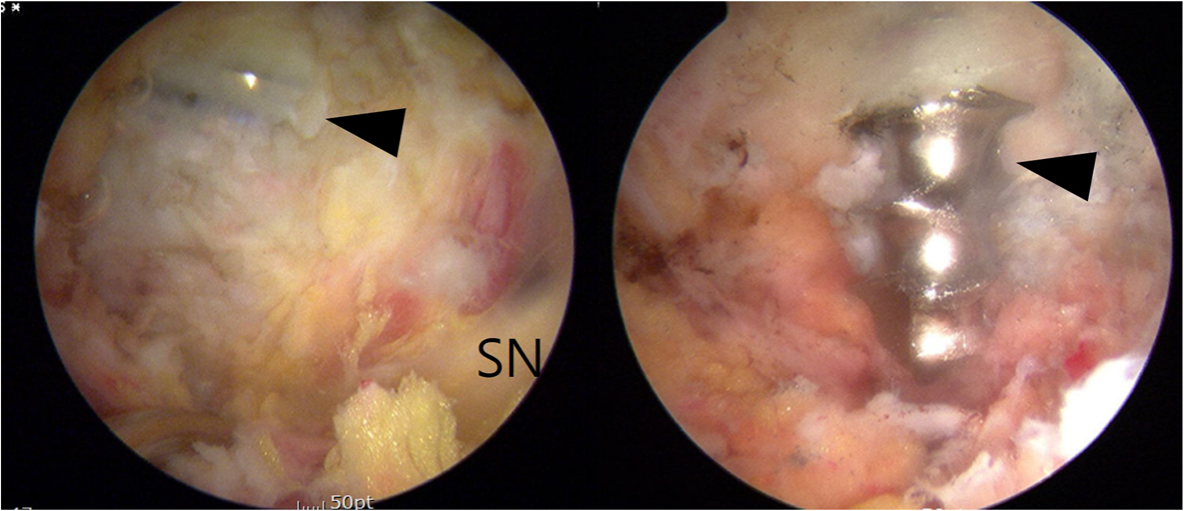
Endoscopic view from anterolateral portal of right hip (supine, ipsilateral upward tilt) showing screw tip (arrow) protruding from posterior column into sciatic notch being irritated sciatic nerve (SN)

**Fig. 7:**
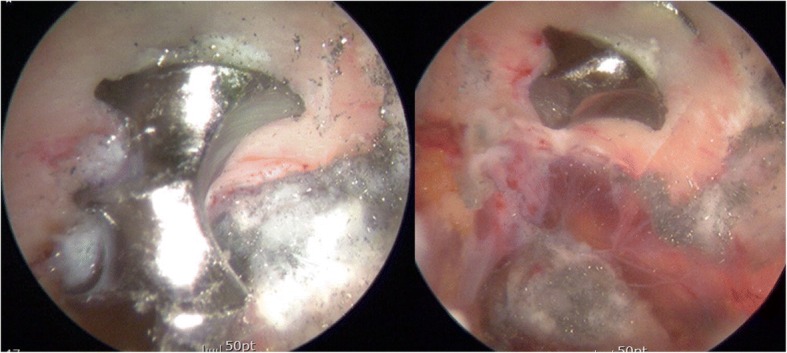
Endoscopic view from anterolateral portal of right hip (supine) showing an acetabular screw being cut proximally by arthroscopic burr

**Fig. 8:**
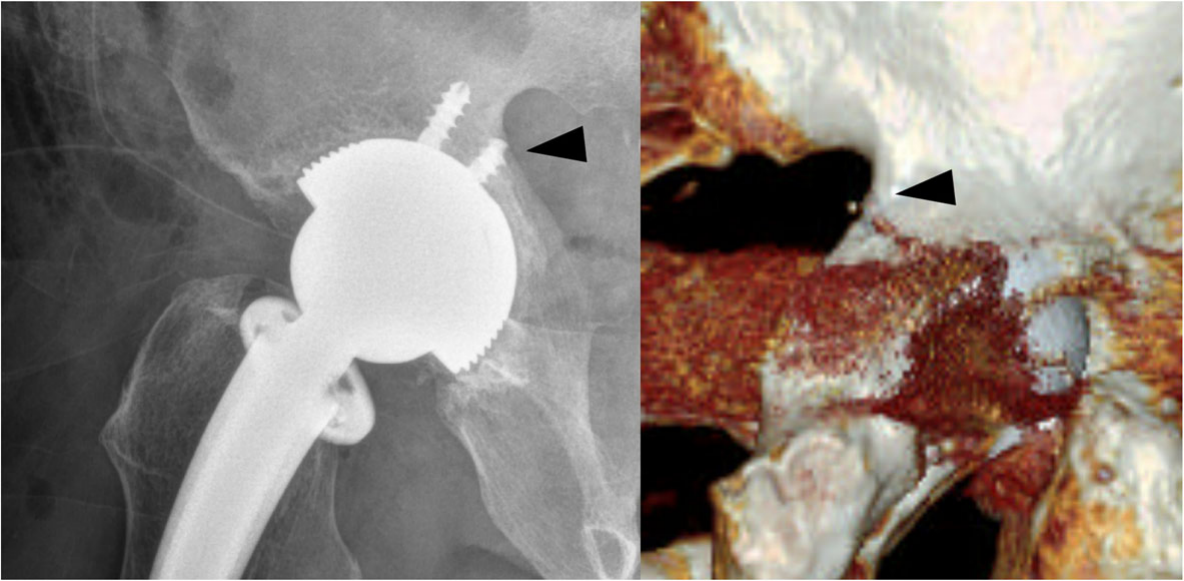
Postoperative radiography and 3-dimensional computed tomography reconstruction showing the resected acetabular screw at sciatic notch level

Eight months after the aforementioned surgery, the patient underwent supine hip arthroscopy without distraction on a fracture table (Hana®, Mizuho OSI). The operating table was tilted right side upward to increase accessibility of ipsilateral buttock. An anterolateral viewing portal and poster lateral working portal were developed without incident. For deep gluteal space visualization, a 70-degree high definition long arthroscope with adjustable length cannulas were utilized. The cannula was opened to maintain the fluid flow when utilizing the radiofrequency probe. Fluid pressure was set to 60 mmHg with intermittent pressure increases up 80 mmHg. After endoscopic greater trochanteric bursectomy, the repaired piriformis from previous revision surgery was well visualized (Fig. 5). Endoscopic visualization of the sciatic nerve revealed a hypo vascular appearance, with entrapped by repaired the piriformis muscle and adjacent fibrous tissue. Endoscopic dynamic testing demonstrated sciatic nerve hypomobility with limited excursion during hip flexion and extension in internal and external rotation. The repaired piriformis tendon was tenotomized and more adjacent scar tissue was observed. Endoscopic resection of fibrovascular bands and adhesions between the piriformis muscle and posterior acetabular wall was performed without incident, allowing visualization of the protruding screw tip penetrated through the piriformis muscle (Fig. 6). Endoscopic piriformis muscle dissection and adhesiolysis was performed, improving visualization of the screw tip adjacent to the supero-lateral aspect of the sciatic nerve. No sciatic nerve intra-substance splitting or tearing was observed, supporting the diagnosis of sciatic nerve irritation without direct nerve injury. Via endoscopic and intermittent multiplanar fluoroscopic visualization, further dissection was performed proximally and distally to prevent inadvertent injuries to the sciatic nerve and the superior gluteal neurovascular bundle. Partial osteoplasty of the sciatic notch with a motorized burr performed under endoscopic guidance. Endoscopic resection of the offending screw tip was performed with a 5.5 mm motorized round burr (Fig. 7). The adjustable cannula protected the adjacent superior gluteal vessels from iatrogenic harm. The screw was scored from the superolateral direction to minimize the risk of unexpected injury by the burr after decorticating the sciatic notch. The protruded screw tip was completely resected and subsequently recontoured to a smooth surface. The screw tip was removed enbloc with an arthroscopic grasper (Fig. 8). Generated metallic debris were removed via suction through the burr cannula. All visible metallic debris was removed. Dynamic arthroscopic and fluoroscopic examinations confirmed successful decompression of the acetabular screw with an immediate visible improvement in sciatic nerve vascularity and excursion. Key procedural steps of this arthroscopic procedure are shown as Additional file 1: Endoscopic technique.

After this endoscopic surgery, immediate hip range of motion was begun with a continuous passive motion machine. The pre-operative pain and paresthesias resolved post-operatively after recovering from anesthesia. The patient advanced to household ambulation without upper extremity aids at 1 postoperative week despite recommendations for 2 weeks of ambulation using crutches. By 3 months postoperatively, he had returned to full activities. At 6 months postoperatively, he had a negative active and passive piriformis test and he remained pleased with the outcome. Postoperative imaging included radiographs and computed tomography scans with 3-dimensional reconstruction demonstrating complete resection of the offending portion of the acetabular screw.

## Discussion

Nerve lesions following total hip arthroplasty (THA) are disabling complications occurring in 0.06 to 2.2% of arthroplasties [5, 6]. Hardware-induced sciatic nerve neuropathies are rarely reported [1, 6]. If placed in the posterior superior quadrant, the screws may be directed toward the sciatic nerve [7]. Mechanical irritation should be suspected in cases of any sign of sciatic neuropathy after THA. The treatment of nerve injuries is tailored its causation. In most cases, the cause is unknown and treatment is directed toward managing symptoms rather than reversing the nerve injury [8]. Open sciatic nerve exploration with dissection of nerve bundle and burr-decompression of the screw has been reported [9]. A protruding acetabular screw can tether the sciatic nerve, restricting its excursion. Endoscopic resection of an offending acetabular screw has now been described. Indeed, had this protruded screw not been resectable by endoscopic means, open exploration and screw removal or resection may have been required. Some advantages of deep gluteal space endoscopy as a minimally invasive procedure are demonstrated in this case report. Decreasing morbidity with minimal blood loss while avoiding re-revision surgery with hip dislocation was facilitated. Moreover, early joint mobilization, relatively rapid postoperative rehabilitation, and outstanding cosmesis were realized. Surgical skills gained from experience with endoscopic technique for deep gluteal syndrome exploration and comfort with the 70 degree arthroscope aided the performance of this surgery. Careful management of endoscopic fluid pressure minimized risk of iatrogenic fluid extravasation into the intra-abdominal and/or retroperitoneal spaces.

An expandable cannula facilitated safe resection of the protruding screw tip while avoiding injury to the adjacent superior gluteal nerve and sciatic nerve. Circulating endoscopy fluid might decrease thermogenesis during metal-on-metal burring of the offending screw. In addition, generated metallic debris is removed immediately through suction system attached burr and cannula to minimize bodily retention with possible adverse consequences.

The decision to remove rather than try to preserve the piriformis muscle was influenced by the surgeon’s experience with deep gluteal space exploration often performed for sciatic nerve decompression.

Relevant suggestions and pearls are summarized in Table 1.

**Table 1:** Pearls for endoscopic resection of protruded acetabular screw irritating sciatic nerve

Perform preoperative assessment of feasibility approaching the location of screw. Perform accurate preoperative self-assessment of surgical experience and arthroscopic skills. Consider patient position (supine or lateral) allowing for manual manipulation of the lower limb at the knee and hip joints for the full assessment of sciatic nerve excursion Lateral position may facilitate ease of conversion to open surgery Prepare for possible open resection of screw or revision total hip arthroplasty (rather than endoscopic resection) if endoscopic method fails. Consider fluoroscopic guidance to identify and confirm resection of the protruding screw. Mobilize and development of soft tissue around sciatic notch. Consider sciatic notch osteoplasty to expose proper cutting level of the screw. Pay careful attention to safe position of burr to prevent superior gluteal neurovascular bundle and sciatic nerve injury (may require several accessory portals). Circulating fluid during burr resection of screw may minimize thermogenesis and metallic debris retention. Confirm adequate resection and smooth recontour of the screw by arthroscopic dynamic testing while envisioning the sciatic nerve Allow early mobilization of hip commensurate with symptomatic improvement.	

For this patient, resection of offending screw tip using endoscopy was considered first, because the acetabular component implanted revision surgery had bony ingrowth without evidence of loosening. Although sciatic nerve irritation from a protruding acetabular screw is rare and its described treatment even rarer, the endoscopic techniques described herein may have broader applications. Recent interest in hip arthroscopy along with more advanced techniques gained from the endoscopic management of sciatic nerve entrapment syndrome/deep gluteal syndrome have allowed the application of minimally invasive hip surgery for conditions once thought treatable only by open sciatic nerve exploration, screw resection and/or revision hip arthroplasty. The endoscopic exploration and screw resection described herein enables less invasive surgery permitting early joint motion, accelerated rehabilitation, and potential outpatient management. Beyond iliopsoas tenotomy, endoscopic treatment may expand to other complication of THA including protruding screws causing adjacent neurovascular compromise.

## Conclusion

A protruding acetabular screw at the sciatic notch was decompressed by use of techniques gained from experience performing endoscopic sciatic nerve decompression. Expandable cannula was used to protect superior gluteal neurovascular bundle proximally and sciatic nerve distally, followed by exposure of screw using an arthroscopic shaver.

## Additional file

Additional file 1: Endoscopic section of acetabular screw tip to decompress sciatic nerve. Via endoscopic and intermittent multiplanar fluoroscopic visualization, further dissection was performed proximally and distally to prevent inadvertent injuries to the sciatic nerve and the superior gluteal neurovascular bundle. Partial osteoplasty of the sciatic notch with a motorized burr performed under endoscopic guidance. Endoscopic resection of the offending screw tip was performed with a 5.5 mm motorized round burr. (MP4 4460 kb)

## Abbreviation

THA: Total hip arthroplasty
